# Comparison of the Application of Three Methods for the Determination of Outdoor PM_2.5_ Design Concentrations for Fresh Air Filtration Systems in China

**DOI:** 10.3390/ijerph192416537

**Published:** 2022-12-09

**Authors:** Xin Zhang, Hao Sun, Kaipeng Li, Xingxin Nie, Yuesheng Fan, Huan Wang, Jingyao Ma

**Affiliations:** 1 School of Resources Engineering, Xi’an University of Architecture and Technology, Xi’an 710055, China; 2 School of Environmental and Municipal Engineering, Xi’an University of Architecture and Technology, Xi’an 710055, China; 3 School of Building Services Science and Engineering, Xi’an University of Architecture and Technology, Xi’an 710055, China

**Keywords:** fresh-air filtration systems, PM_2.5_, no-guarantee days, guarantee rate, mathematical inductions

## Abstract

With the increasing popularity of fresh-air filtration systems, the methods of determining the outdoor PM_2.5_ design concentration have become more important. However, the monitoring of atmospheric fine particles in China started relatively late, and there are relatively few cities with complete data, with obvious regional differences, which led to many problems in the selection of air filters for fresh-air filtration systems. In this paper, three methods of determining outdoor PM_2.5_ design concentration were analyzed using the daily average concentration of PM_2.5_ in 31 provincial capital cities from 2016 to 2020. Six typical cities in different regions were also taken as examples. The advantages and disadvantages of the three existing statistical methods were compared and analyzed, as well as the corresponding differences in the selection of outdoor PM_2.5_ concentration value on the filter systems. The results showed that the method of mathematical induction was more accurate and reasonable for the calculation of outdoor PM_2.5_ design concentrations. The local outdoor PM_2.5_ design concentration could be quickly calculated using the recommended coefficient *K* and annual average PM_2.5_ concentration of the region, especially for small and medium-sized cities without monitoring data. However, the recommended coefficient *K* should be provided based on the specific region, and should be divided into values for strict conditions and normal conditions during use. This would provide a simple and effective way to select the correct air filters for practical engineering.

## 1. Introduction

With the increasing concentrations of particulate matter in the atmosphere, how to build a good indoor environment is a current focus of attention. Haze weather frequently appears in most cities in China [1,2,3], especially in winter [3]. In addition, PM_2.5_ in the atmosphere might cause some diseases related to human lung disease, cardiovascular disease, etc. [4,5,6,7,8]. However, people spend as much as 80–90% of their time indoors [9], and the quality of the indoor environment was the most important to us. The relevant literature showed that ~50% of indoor PM came from the outdoor environment [10]. Outdoor air pollution enters the room through the ventilation systems and gaps in building envelopes [11]. With the outbreak of Coronavirus Disease 2019 (COVID-19) [12], complex cross-contamination in the environment brought greater challenges and had an impact on the indoor environment. Meanwhile, with the continuous improvement in buildings’ airtightness levels [13], indoor environments have become relatively closed. As a result, the fresh-air system has become an important way to effectively solve the indoor sanitary environment issue and provide fresh air [14].

The design and selection of the air filters in fresh-air systems determines the filtration performance of air pollutants [15]. The overall efficiency of the air filters was calculated using the design concentration value of outdoor PM_2.5_ and the design concentration value of indoor PM_2.5_. The design concentration value of outdoor PM_2.5_ is an important parameter for air-filter selection [16]. At present, there is no national standard to provide an exact outdoor PM_2.5_ design concentration value in China, and there is no provision for the corresponding outdoor PM_2.5_ design concentration method [16]. As a result, there is no definite value for the design parameters of air filters during the design selection process, and the indoor air quality does not meet the standard.

Many researchers from different countries have conducted related studies. Some of the related research was on filter materials, such as ways to improve filtration efficiency [17], reduce the cost of filter materials [18], and develop new materials [19,20,21]. Some achievements have been made, but there is still a difference between filter selection and use, which cannot provide a stable and safe form of indoor environmental hygiene. The main reason for this might be that the design parameters during the selection process were different from the actual situations.

Some scholars conducted related research on the design concentrations of outdoor particulate matter [15,22,23]. They proposed and verified various determination methods, such as no-guarantee days [6,15], guarantee rate [22], and no-guarantee hours [23]. Although those methods brought certain improvements, the selection of fresh-air filters is still slightly insufficient. The main reasons for this are that the monitoring of outdoor PM_2.5_ started late in China, there is no unified determination method at present, and the statistical time and methods of calculating parameters also have not been studied in depth [24]. In addition, the concentrations of outdoor atmospheric particulate matter are always variable, with regional characteristics [25]. They are also related to the local energy structures and industrial chains, and the concentration distributions of particulate matter also greatly vary in various districts of the same city. The existing methods are based on the results of provincial capital cities, using monitoring data. For small and medium-sized cities, it is difficult to calculate the outdoor design concentration without a complete monitoring system. Comparisons and analyses of existing methods are also lacking, as well as a summary of their advantages, disadvantages and adaptability.

In addition, the source distributions of PM_2.5_ were more complex, but some existing studies directly applied foreign determination methods, without conducting an in-depth examination of their applicability, which caused large differences in the values of outdoor design concentrations in different regions and affected the indoor environment [26]. For example, the value of the outdoor design concentration was too small in areas with heavier pollution, which might result in the indoor air quality not meeting the standards and endangering human health, while its value was too large in areas with lighter pollution, which might increase the initial investment, operation and maintenance costs of equipment, causing unnecessary waste [16]. Cities in different provinces also need to provide different outdoor design concentration values according to their actual conditions, and the design concentration values of various regions cannot be confused.

The methods of determining the outdoor PM_2.5_ design concentration for fresh-air filtration systems underwent an in-depth comparison and analysis in this paper, based on existing research results in China. Various factors and the actual situation were comprehensively analyzed to provide a relatively more reasonable method of determining the outdoor PM_2.5_ design concentration. Reference values are provided for the selection of air filters, and some suggestions to control air pollution are given, with strong practical engineering application significance.

## 2. Methods

### 2.1. Data Sources

The data in this paper were from related networks, such as http://www.tianqihoubao.com/aqi/xian.html, accessed on 6 June 2021 and http://www.tianqi.com/air/xian.html, accessed on 6 June 2021. The hourly average concentration values for PM_2.5_ in the atmosphere from 31 January 2016 to 31 December 2020 were used.

### 2.2. The Method for No-Guarantee Days

The method for no-guarantee days used the outdoor calculated concentration of PM_2.5_ to adopt an average daily mass concentration that was not guaranteed for specific days [6,15,22]. For example, if the daily mass concentration was not guaranteed for 5 days, the average mass concentration of PM_2.5_ per year in each statistical year should be arranged in descending order, and then the highest 5 days in terms of average mass concentration of PM_2.5_ should be removed. The average mass concentration of PM_2.5_ on the sixth day is the daily mass concentration that is not guaranteed for 5 days [6,15,22]. No-guarantee for 10 days means that the average mass concentration of PM_2.5_ per year in each statistical year should be arranged in descending order, and then the highest average mass concentration of PM_2.5_ for 10 days should be removed. The average mass concentration of PM_2.5_ on the 11th day is the daily mass concentration that is not guaranteed for 10 days.

### 2.3. The Method of Guarantee Rate

The method of guarantee rate creates a guarantee rate curve according to the corresponding outdoor PM_2.5_ design concentration. The corresponding outdoor design concentration value is determined according to the required guarantee rate [22].

Firstly, the PM_2.5_ concentration grouping distance is calculated, as well as the number of groups and upper limit between different groups of PM_2.5_ concentration in different cities. The calculation formulas are as follows in Equations (1) and (2) [22]:

(1) $$C_{n}=\frac{C_{\max}-C_{\min}}{n}\approx C$$

(2) $$C_{i}=i\times C$$

where *C_n_* is the concentration group interval group distance; *C*_max_ and *C*_min_ are the maximum and minimum concentrations of PM_2.5_ in a recorded time period, respectively, μg/m^3^; *n* is the number of groups, generally 5–10; *C* is the integer value greater than and closest to *C_n_*; *C_i_* is the upper limit of the group.

Secondly, counting the frequency of different groups, and the number of days that the PM_2.5_ concentration values appear between different groups, the relative frequency of each group is calculated using Equation (3) [22]:

(3) $$f_{i}=\frac{N_{i}}{\sum_{i}^{n}N_{i}}\times 100\%$$

where *f_i_* is the relative frequency of group *i*, %, and *N_i_* is the frequency of group *i*.

Thirdly, the cumulative relative frequency corresponding to each grouping interval is calculated in the grouping order from small to large using Equation (4) [22], which shows that the cumulative relative frequency of group *i* is equal to the sum of the relative frequencies of *i* groups:

(4) $$F_{i}=\sum_{i}^{n}f_{i}$$

where *F_i_* is the cumulative relative frequency of group *i*, %.

Finally, the cumulative relative frequency is shown as the horizontal axis, while the upper limit of PM_2.5_ concentration group is shown as the vertical axis [22]. The cumulative relative frequency is the guarantee rate. The PM_2.5_ concentration corresponding to the guarantee rate is the outdoor PM_2.5_ design concentration value.

### 2.4. The Method of Mathematical Induction

The concentrations of particulate matter in the outdoor atmosphere are affected by many factors, and the law of changes is also relatively complicated. Therefore, professionals in Japan conducted an inductive analysis, and summarized this in six levels of “risk rates” (non-guaranteed rates). The PM_10_ concentration values matched the risk rates, which were 2.5%, 5%, 10%, 15%, 20%, and 50%, respectively [16,24]. The regression correlations between the concentration values of atmospheric particulate matter and the annual average values at different guarantee rates were obtained [16,24]. The design concentrations of atmospheric particulate matter *C_D_* in each area were calculated using Equation (5) [16,24]:

(5) $$C_{D}=KC_{y}$$

where *C_y_* is the annual average concentration of suspended particulate matter in the atmosphere in the area; *K* is the recommended coefficient. The recommended coefficient *K* is the ratio of the annual average value to the outdoor PM_2.5_ design concentration value corresponding to different non-guaranteed rates [16,24].

This method could be used for situations where the monitoring data are relatively few, equipment is relatively imperfect, and data are lacking. Therefore, it is more in line with the current basic national conditions in China [16].

### 2.5. The Method of Air Filter Selection

Particulate concentrations in indoor environments are determined by filtration efficiency, and air filters are selected according to their filtration performance and the different grades of fresh-air filtration systems. The efficiency of air filters can be calculated by Equation (6) [16]:

(6) $$\eta =\frac{C_{1}-C_{2}}{C_{1}}\times 100\%$$

where *η* is the filtration efficiency of air filters, %; *C*_1_ is the outdoor PM_2.5_ design concentration, μg/m^3^; *C*_2_ is the indoor PM_2.5_ design concentration, μg/m^3^.

The series’ combined efficiency could be calculated using Equation (7):

(7) $$\eta =1-(1-\eta_{1})(1-\eta_{2})(1-\eta_{3})\cdots (1-\eta_{n})$$

where *η* is the series combined efficiency of air filters, %; *η*_1_ to *η_n_* is the filtration efficiency of each filter grade, %.

## 3. Results and Discussion

### 3.1. Outdoor Concentration of PM_2.5_

**Figure 1:**
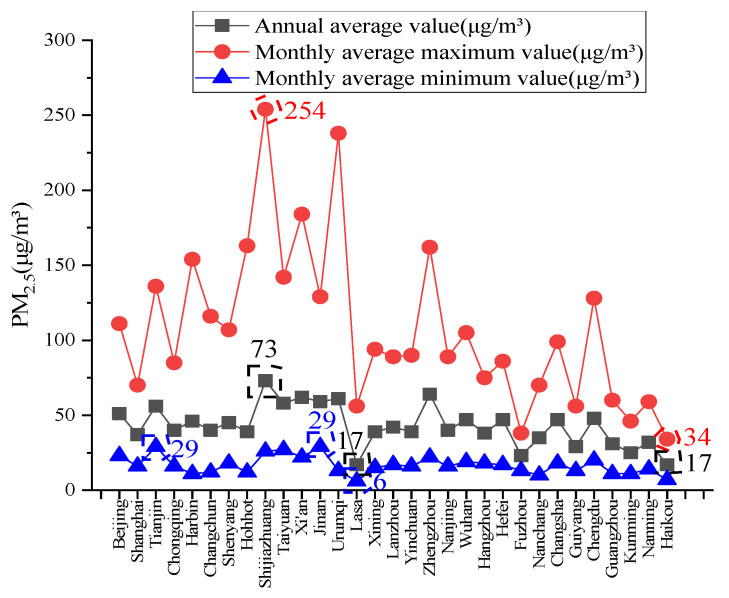
Average concentration of PM_2.5_ in major cities of China from 2016 to 2020.

The PM_2.5_ values in 31 major cities in China from 2016 to 2020 are shown in Figure 1.

Figure 1 shows that the annual average concentration of PM_2.5_ in 31 cities in China from 2016 to 2020 was 17~73 μg/m^3^. The cities with the smallest 5-year average concentration of PM_2.5_ were Lasa and Haikou, while the largest was Shijiazhuang. Among the 31 cities, the city with the highest monthly average concentration of PM_2.5_ was Shijiazhuang, which appeared in December 2016, with 254 μg/m^3^. This was followed by Urumqi, Xi’an, Hohhot and Zhengzhou, where the highest concentrations appeared in January 2017, January 2017, January 2020, and December 2016, respectively. The city with the lowest monthly average concentration of PM_2.5_ was Lasa, at 6 μg/m^3^. The outdoor concentrations of PM_2.5_ in China were unevenly distributed: the PM_2.5_ concentration in southern coastal cities was relatively low, while that in northern cities was relatively high. Therefore, an in-depth study of the method used to determine the outdoor PM_2.5_ concentration was significant to provide a reference for the accurate and reasonable selection of fresh-air filtration systems. The concentration control limit for indoor PM_2.5_ could be determined according to the relevant air-quality control standards [27].

### 3.2. The Change in PM_2.5_ Concentration Using the Method of No-Guarantee Days

The monitoring data for 31 provincial capitals in China, from 2016 to 2020, were counted and analyzed based on the existing relevant research and conclusions. The outdoor atmospheric PM_2.5_ concentration values, corresponding to the different not-guaranteed day parameters, are given in Table 1 [6,15].

**Figure 2:**
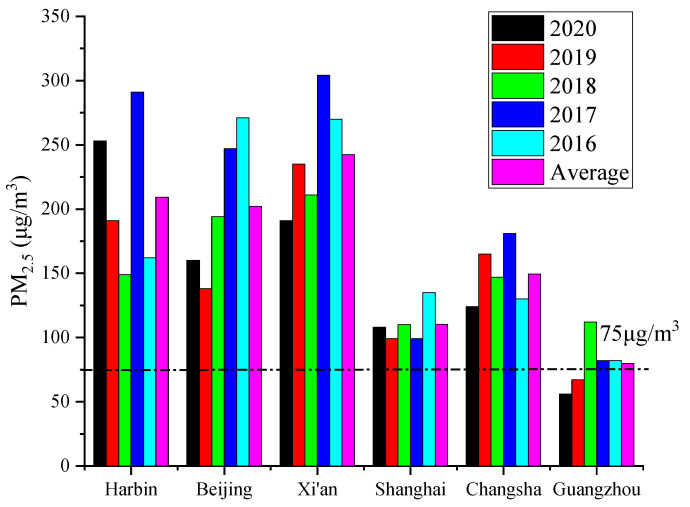
The outdoor concentration value corresponding to no-guarantee for 5 days.

Table 1 shows that the outdoor PM_2.5_ concentration values corresponding to situations of no-guarantee for 5 days and no-guarantee for 10 days in each city within 5 years were very different. With an increase in the number of no-guarantee days, the corresponding outdoor PM_2.5_ concentration values gradually decreased. The maximum concentration difference corresponding to no-guarantee for 5 days and no-guarantee for 10 days in the whole of 2020 was 54 μg/m^3^, and the corresponding cities were Tianjin and Harbin; the minimum concentration difference was 4 μg/m^3^, and the corresponding cities were Lasa, Lanzhou, Chengdu, and Kunming. The maximum concentration difference in 2019 was 42 μg/m^3^, and the corresponding city was Shenyang; the minimum concentration difference was 2 μg/m^3^, and the corresponding city was Lasa. The maximum concentration difference in 2018 was 33 μg/m^3^, and the corresponding city was Jinan; the minimum concentration difference was 4 μg/m^3^, and the corresponding cities were Guiyang and Kunming. The maximum concentration difference in 2017 was 70 μg/m^3^, and the corresponding city was Zhengzhou; the minimum concentration difference was 3 μg/m^3^, and the corresponding city was Lasa. The maximum concentration difference in 2016 was 152 μg/m^3^, and the corresponding city was Zhengzhou; the minimum concentration difference was 2 μg/m^3^, and the corresponding city was Nanning. Therefore, a more demanding living environment could be obtained using no-guarantee for 5 days. In addition, using data for many years led to more accurate results [6,15]. For further analysis, the comparison results of the outdoor concentration values of six typical cities, corresponding to no-guarantee for 5 days, are shown in Figure 2. The six representative cities, with obvious regional differences, were Harbin (longitude: 125°42′ to 130°10′ E, latitude: 44°04′ to 46°40′ N), Beijing (longitude: 115°25′ to 117°30′ E, latitude: 39°26′ to 41°03′ N), Xi’an (longitude: 107°40′ to 109°49′ E, latitude: 33°42′ to 34°45′ N), Shanghai (longitude: 120°52′ to 122°12′ E, latitude: 30°40′ to 31°53′ N), Changsha (longitude: 111°53′ to 114°15′ E, latitude: 27°51′ to 28°41′ N), and Guangzhou (longitude: 112°57′ to 114°30′ E, latitude: 22°26′ to 23°56′ N), respectively.

Figure 2 shows that the outdoor concentration values for each year, corresponding to no-guarantee for 5 days, were quite different. The PM_2.5_ concentration values would rebound to a certain extent; the pollution was heavier or lower than the previous year. Only the outdoor PM_2.5_ concentration values for Guangzhou in 2020 and 2019 were lower than the standard (75 μg/m^3^) [28], at 56 μg/m^3^ and 67 μg/m^3^, respectively. The differences between the maximum value and the minimum value in 5 years for Harbin, Beijing, Xi’an, Changsha, Guangzhou, and Shanghai were 142 μg/m^3^, 133 μg/m^3^, 113 μg/m^3^, 57 μg/m^3^, 56 μg/m^3^ and 36 μg/m^3^, respectively. The largest difference between the outdoor concentration in each year and the 5-year average concentration was found for Harbin, with 81.8 μg/m^3^. The smallest difference was found for Guangzhou, with 2.2 μg/m^3^. Therefore, it could be seen that the outdoor PM_2.5_ design concentration, calculated using the method of no-guarantee days, still had certain fluctuations. The method of calculating no-guarantee days using the average data for many years was more reasonable. Related research also gave the same results [6,15], which again verified the correctness of this paper. However, a large amount of data are needed for analysis if the method of no-guarantee days uses the data for many years. In addition, the statistical analysis process is relatively cumbersome, requiring extensive time to complete, and errors occur in the data processing.

### 3.3. The Change in PM_2.5_ Concentration Using the Method of Guarantee Rate

**Figure 3:**
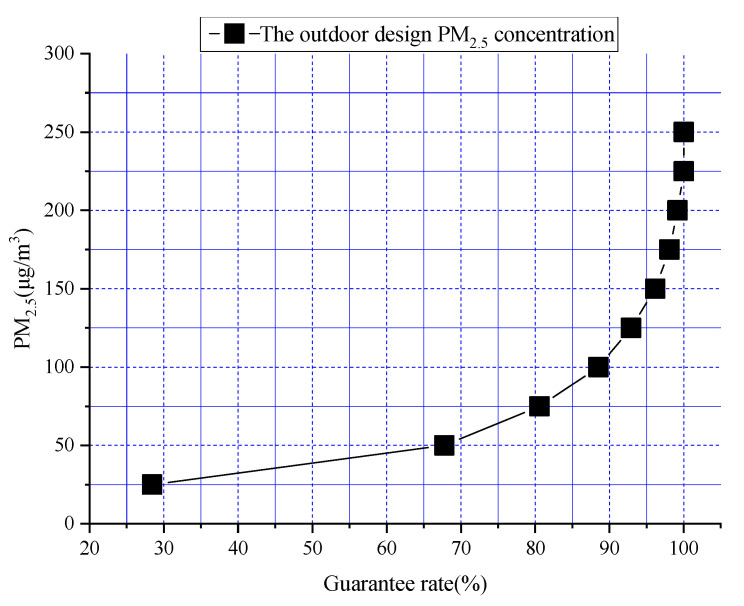
Guarantee rate curve for Xi’an.

Xi’an was taken as an example to draw a guarantee-rate curve to increase understanding of the guarantee rate method. The daily average PM_2.5_ concentration values for the whole year of 2020 were sorted, and the maximum and minimum values were 225 μg/m^3^ and 6 μg/m^3^, respectively. The difference between them was 219 μg/m^3^, *n* was 10, *C_n_* was 21.9 (Equation (1)), and *C* was 25. The group limits were 25, 50, 75, 100, 125, 150, 175, 200, 225, and 250 (Equation (2)), and the total number of days was 366. The calculation results for the relative frequency (Equation (3)) and the cumulative relative frequency (Equation (4)) are shown in Table 2. A guarantee rate curve for Xi’an for the whole of 2020 is shown in Figure 3.

Figure 3 shows the change trend for the outdoor PM_2.5_ concentration of the guarantee rate and its specific values. If the guarantee rate was 95%, the outdoor PM_2.5_ design concentration was about 133 μg/m^3^. If the guarantee rate was 97.5%, the outdoor PM_2.5_ design concentration was about 163 μg/m^3^. The data for a total of 5 years, from 2016 to 2020, were used for calculation using the method, and the outdoor PM_2.5_ design concentrations of different guarantee rates for 31 major cities in China could be obtained. The outdoor PM_2.5_ design concentration values corresponding to guarantee rates of 95% and 97.5% for six typical cities are shown in Table 3.

The outdoor PM_2.5_ concentration values also had a certain rebound phenomenon when the guarantee rates were 97.5% and 95%. The higher the guarantee rate, the greater the concentration rebound. Only the outdoor PM_2.5_ concentration values corresponding to a guarantee rate of 95% in Guangzhou were lower than the standard (75 μg/m^3^) [28]. Except for 2019, the outdoor PM_2.5_ concentration values corresponding to a guarantee rate of 97.5% in Guangzhou were lower than the standard (75 μg/m^3^) [28]. Except for 2016, the outdoor PM_2.5_ concentration values corresponding to a guarantee rate of 95% in Shanghai were lower than the standard (75 μg/m^3^) [28]. The largest difference between the outdoor concentration in each year and the 5-year average concentration was found in Beijing, at 87.6 μg/m^3^, while the smallest difference was found in Guangzhou, at 0.8 μg/m^3^. However, the selection of the specific guarantee rate was related to many factors, such as the types of buildings, their specific use requirements, the local environment, etc., as well as the needs of designers. The outdoor PM_2.5_ design concentration of any guarantee rate could be obtained according to the drawn guarantee rate curve. Therefore, the required values could be easily found for the guarantee rate of 0–100% [22].

### 3.4. The Change in PM_2.5_ Concentration Using the Method of Mathematical Induction

**Figure 4:**
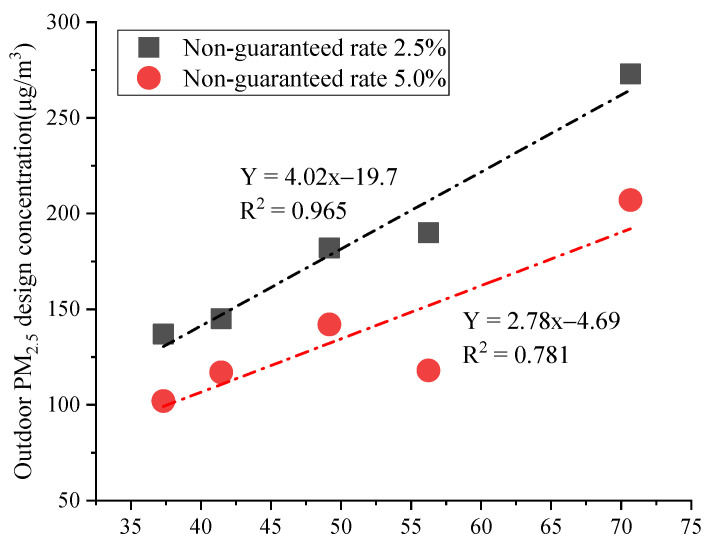
Relationship between the annual average values and the design concentration of PM_2.5_.

Beijing was taken as an example to facilitate understanding of the mathematical induction method. The annual average values of PM_2.5_ concentration and their average concentration value, corresponding to non-guaranteed rates of 2.5% and 5.0%, were calculated using monitoring station data from 2016 to 2020. The relationship between the annual average values of PM_2.5_ concentration and their average concentration value is shown in Figure 4, in which the average concentration values correspond to non-guaranteed rates of 2.5% and 5.0%.

**Figure 5:**
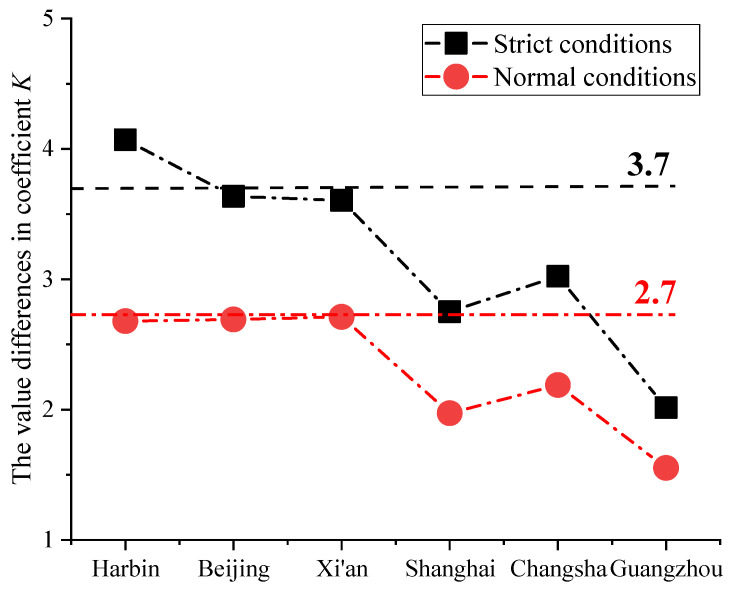
Differences in coefficient *K* between China and Japan.

Figure 4 shows that the fitting curves had good fitting effects for the non-guaranteed rates of 2.5% and 5%. The outdoor PM_2.5_ design concentration values for different cities in China also could be calculated using the method of mathematical induction. However, China is relatively vast, and the source distribution of PM_2.5_ in different regions is quite unbalanced. The recommended coefficient *K* for Japan cannot be directly applied in China, and the same recommended coefficient *K* could not be adopted for the whole country [16]. The recommended coefficient *K* for Japan is usually divided into values for strict conditions and normal conditions. For strict conditions, the non-guaranteed rate was 2.5%, and the recommended coefficient *K* was 3.7. For normal conditions, the non-guaranteed rate was 5% and the recommended coefficient *K* was 2.7 [24]. Strict conditions refer to buildings with extremely high requirements for the concentration of indoor particulate matter, such as clean rooms and wards and dust-free workshops. Normal conditions refer to environments that do not need to strictly control the concentration of indoor particulate matter, such as houses, shopping malls, schools, and airports [16]. The differences between the recommended coefficient *K* for Japan and six typical cities in China are shown in Figure 5.

Figure 5 shows that the average recommended coefficient *K* for each city in China was quite different, and there was a big difference with the *K* value recommended for Japan. Among the six typical cities in China, only the recommended *K* for Harbin was higher than that for Japan under strict conditions, while only the recommended *K* for Xi’an was higher than that for Japan under normal conditions. Therefore, the recommended *K* for Japan could not be directly applied in China. The maximum value of coefficient *K* was found in Harbin under the non-guaranteed rate, at 2.5%, while the average value was 4.07. The minimum value of coefficient *K* was found in Guangzhou; the average value was 2.02, and the difference between the two cities was 2.05. The maximum value of coefficient *K* was found in Xi’an under the non-guaranteed rate, at 5%, with an average value of 2.71. The minimum value of coefficient *K* was still found in Guangzhou; the average value was 1.55, and the difference between Guangzhou and Xi’an was 1.16. The largest difference in the coefficient *K* value in the same city was found in Harbin, at 1.39, while the smallest difference was found in Guangzhou, at 0.463. Therefore, it could be seen that the same recommended coefficient *K* could not be used. The recommended coefficient *K* should be provided separately according to region. Different provinces and cities need different coefficient *K* recommendations. In addition, there is a new trend under way in the regional economies of China at present that differentiates north–south economic growth, and the development of the south’s economy is rapid relative to that of the north [29]. Therefore, there will be greater gaps between the values of coefficient *K* for different cities. The recommended coefficient *K* values for six typical cities in China from 2016 to 2020 under strict conditions (non-guaranteed rate 2.5%) and normal conditions (non-guaranteed rate 5%) are shown in Table 4.

Therefore, the outdoor design concentration values could be obtained according to the recommended coefficient *K* and the annual average concentration values. Based on the development status of China, there were relatively few atmospheric PM_2.5_ concentration data values available for this analysis, so the method of mathematical induction was an applicable and simple method to calculate the outdoor PM_2.5_ design concentrations.

### 3.5. Analysis and Suggestions Regarding the Calculation Results of Three Methods

The outdoor PM_2.5_ design concentrations, calculated according to three existing methods, are summarized in Table 5.

**Figure 6:**
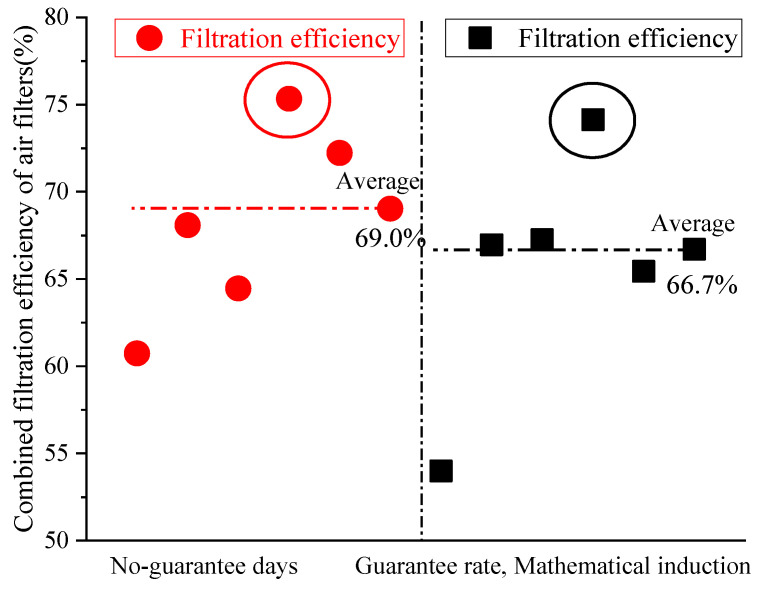
Comparison of outdoor PM_2.5_ concentration determination methods for fresh-air filtration.

Table 5 shows that the calculation results for the three methods were very different, and the concentration value obtained using the method of no-guarantee days was the highest. The 5-year average outdoor concentrations of Harbin, Beijing, Xi’an, Shanghai, Changsha and Guangzhou, calculated using the method of no-guarantee days, were 23.2 μg/m^3^, 16.6 μg/m^3^, 17 μg/m^3^, 9.2 μg/m^3^, 6.6 μg/m^3^ and 18 μg/m^3^ higher than those obtained using the other two methods, respectively. The largest difference was found in Harbin, and the smallest difference was found in Changsha. The difference in Beijing in 2017 was the largest when using a single year’s data for calculation and comparison, at 57 μg/m^3^. The second largest difference was found in Xi’an in 2016, at 53 μg/m^3^. It is recommended to use the average value of previous years for calculation, to ensure results are more stable and accurate. Xi’an was taken as an example for a comparative analysis of the differences using the three methods. The outdoor PM_2.5_ concentrations were calculated using the data in Table 5, and the indoor concentration was 75 μg/m^3^ [28]. The comparison of methods for the determination of outdoor PM_2.5_ concentrations for fresh air filtration methods in Xi’an is shown in Figure 6.

Figure 6 shows that the filtration efficiency of air filters ranged from 60.7% to 75.3% using the method of no-guarantee days, and the average efficiency was 69.0%. The filtration efficiency of air filters ranged from 54.0% to 74.1% using the methods of guarantee rate and mathematical induction, and the average efficiency was 66.7%. The results obtained using the method of no-guarantee days were higher than those obtained using the methods of guarantee rate and mathematical induction. The relevant parameters of existing air filters of different grades, according to previous market research and related test research, are given in Table 6 [16,30,31].

The series combination of air filters is given in Table 7 based on the performance parameters, which met the indoor air quality standards of Xi’an from 2016 to 2020. The relevant filter equipment could be quickly adjusted and selected according to this table.

According to a comprehensive comparative analysis of the above three methods, the method of no-guarantee days showed relatively large changes, which might increase the initial investment in equipment. The variances in outdoor PM_2.5_ concentration obtained using the guarantee rate and mathematical induction methods were relatively small. The basic principles of the two methods of no-guarantee days and guarantee rate are the same. The outdoor PM_2.5_ design concentration of any guarantee rate could be obtained according to the guarantee rate curve drawn using the guarantee rate method. In addition, the guarantee rate method is more complicated than the method of no-guarantee days, with relatively more steps. Although both methods have been applied in practice, the guarantee rate method was used more often, for more days [32].

The method of mathematical induction was based on a large amount of statistical data on induction and analysis, and the outdoor PM_2.5_ design concentration could be quickly and easily calculated according to the recommended constant *K* and the annual average values. This method is more appropriate, especially for the outdoor PM_2.5_ monitoring work that started relatively late in China, for and outdoor PM_2.5_ pollution with random, time-varying and regional characteristics. For some small cities, the estimated values for local outdoor PM_2.5_ design concentration could be quickly and easily calculated according to the specific recommended coefficient *K* and the annual average values for the region [16]. The recommended coefficient *K* was given based on specific regions, and the geographical conditions, topography and people’s living customs in these regions were given comprehensive consideration. Therefore, the recommended *K* in a given region can represent the recommended *K* for a city in that region by default. The recommended *K* was provided by calculating the average of the existing monitoring data for provincial capital cities. This effectively overcame the current disadvantage of having relatively few atmospheric PM_2.5_ concentration data values in China available for analysis.

## 4. Conclusions

The methods of no-guarantee days, guarantee rate, and mathematical induction were used in this paper to analyze the daily average concentration of PM_2.5_ in 31 provincial capital cities in China from 2016 to 2020. The advantages and disadvantages of the three existing statistical methods were compared and analyzed, as well as the corresponding differences in the selection of outdoor PM_2.5_ concentration values for filtration systems. The conclusions are given as follows:

1. The results of the method of no-guarantee days showed relatively large changes, and the data were prone to rebound, which leads to large errors when calculating the outdoor PM_2.5_ concentration. The outdoor PM_2.5_ concentration values corresponding to no-guarantee for 5 days were obtained under the strict requirements of the environment. The outdoor PM_2.5_ concentration values corresponding to no-guarantee for 10 days were obtained under the normal requirements of the environment. It is recommended to use the average data over a period of years to increase accuracy.
2. The required outdoor PM_2.5_ design concentration value of any guarantee rate for each city could be obtained according to the guarantee rate curve that is drawn. This satisfies the requirements for environmental control under different guarantee rates. In addition, the guarantee rate was more complicated and used more steps.
3. When the method of mathematical induction is used, the recommended coefficient *K* should be given separately, according to the region. Different provinces and cities need different recommendations for coefficient *K*. The recommended coefficient *K* could be divided into values for strict conditions and normal conditions in practice.
4. It is more appropriate to use the method of mathematical induction to calculate the outdoor PM_2.5_ design concentration for fresh-air filtration. The outdoor PM_2.5_ design concentration in a given location can be quickly obtained using the values of *K* and annual average concentration. The recommended *K* is obtained by calculating the average of the existing monitoring data for provincial capital cities. This could effectively solve the current limited availability of urban atmospheric PM_2.5_ concentration data values for analysis, making this method more suitable for use in China. It provides a reference value for the method to quickly determine the outdoor PM_2.5_ design concentration for fresh-air filtration systems.

## Figures and Tables

**Table 1:** The outdoor calculated concentrations of PM_2.5_ in 31 cities.

City	No-Guarantee for 5 Days						No-Guarantee for 10 Days					
	2020	2019	2018	2017	2016	Average	2020	2019	2018	2017	2016	Average
Beijing	160	138	194	247	271	202	119	123	167	185	244	167.6
Shanghai	108	99	110	99	135	110.2	84	88	98	92	121	96.6
Tianjin	201	174	146	204	256	196.2	147	152	119	184	223	165
Chongqing	90	111	104	145	140	118	79	102	92	131	108	102.4
Harbin	253	191	149	291	162	209.2	199	166	125	236	140	173.2
Changchun	220	153	91	162	138	152.8	167	134	83	151	122	131.4
Shenyang	158	174	112	146	169	151.8	124	132	100	141	140	127.4
Hohhot	221	150	119	131	127	149.6	182	115	99	119	121	127.2
Shijiazhuang	201	228	218	300	436	276.6	180	202	200	268	309	231.8
Taiyuan	178	173	152	267	240	202	158	148	145	199	188	167.6
Xi’an	191	235	211	304	270	242.2	156	209	190	260	212	205.4
Jinan	166	169	179	228	191	186.6	140	157	146	170	179	158.4
Urumqi	216	200	226	308	338	257.6	185	190	208	282	285	230
Lasa	28	23	43	51	69	42.8	24	21	34	48	65	38.4
Xining	86	95	98	112	119	102	79	79	88	104	108	91.6
Lanzhou	82	96	108	129	126	108.2	78	84	92	115	112	96.2
Yinchuan	125	80	86	123	141	111	113	73	76	106	135	100.6
Zhengzhou	185	213	229	286	366	255.8	146	200	201	216	214	195.4
Nanjing	101	107	167	133	145	130.6	90	104	136	96	128	110.8
Wuhan	108	136	136	160	151	138.2	99	112	127	142	139	123.8
Hangzhou	92	96	116	125	123	110.4	75	89	101	108	111	96.8
Hefei	122	123	143	150	156	138.8	100	109	132	126	136	120.6
Fuzhou	50	54	62	58	68	58.4	43	47	54	54	60	51.6
Nanchang	89	92	82	123	136	104.4	79	84	74	105	120	92.4
Changsha	124	165	147	181	130	149.4	111	143	131	157	121	132.6
Guiyang	64	69	75	84	84	75.2	56	59	71	79	71	67.2
Chengdu	106	119	126	183	147	136.2	102	94	111	172	143	124.4
Guangzhou	56	67	112	82	82	79.8	47	64	91	78	76	71.2
Kunming	58	53	61	66	57	59	54	50	57	54	53	53.6
Nanning	78	83	88	100	84	86.6	62	75	80	89	82	77.6
Haikou	47	44	47	61	51	50	41	40	42	53	45	44.2

**Table 2:** Grouping interval and cumulative relative frequency for Xi’an.

Groups (*i*)	Upper Limit (*C_i_*)	Frequency (*N_i_*)	Relative Frequency (*f_i_*)	Cumulative Frequency	Cumulative Relative Frequency
1	0–25	104	28.42%	104	28.42%
2	25–50	144	39.34%	248	67.76%
3	50–75	47	12.84%	295	80.60%
4	75–100	29	7.92%	324	88.52%
5	100–125	16	4.37%	340	92.90%
6	125–150	12	3.28%	352	96.17%
7	150–175	7	1.91%	359	98.09%
8	175–200	4	1.09%	363	99.18%
9	200–225	3	0.82%	366	100.00%
10	225–250	0	0.00%	366	100.00%

**Table 3:** The outdoor PM_2.5_ design concentrations corresponding to different guarantee rates.

City	Guarantee Rate 97.5%						Guarantee Rate 95%					
	2020	2019	2018	2017	2016	Average	2020	2019	2018	2017	2016	Average
Harbin	204	157	153	250	166	186	122	100	105	159	126	122.4
Beijing	137	145	182	190	273	185.4	102	117	142	118	207	137.2
Xi’an	163	227	229	290	217	225.2	133	188	188	195	143	169.4
Shanghai	92	104	103	71	135	101	60	74	70	51	107	72.4
Changsha	131	153	148	173	109	142.8	102	123	95	122	75	103.4
Guangzhou	43	76	69	60	61	61.8	30	68	51	44	45	47.6

**Table 4:** The recommended coefficient *K* values for six typical cities from 2016 to 2020.

City	2020		2019		2018		2017		2016		Average	
	Strict	Normal	Strict	Normal	Strict	Normal	Strict	Normal	Strict	Normal	Strict	Normal
Harbin	4.413	2.639	4.042	2.575	4.051	2.780	4.444	2.826	3.364	2.553	4.071	2.679
Beijing	3.671	2.733	3.499	2.823	3.701	2.888	3.378	2.098	3.863	2.929	3.637	2.692
Xi’an	3.236	2.641	3.912	3.240	3.765	3.091	3.999	2.689	3.075	2.026	3.605	2.712
Shanghai	2.955	1.927	3.035	2.159	2.902	1.972	1.855	1.332	3.040	2.410	2.751	1.972
Changsha	3.220	2.507	3.289	2.644	3.277	2.103	3.358	2.368	2.083	1.433	3.023	2.189
Guangzhou	1.916	1.337	2.581	2.309	2.073	1.532	1.761	1.291	1.794	1.323	2.016	1.553

**Table 5:** Outdoor PM_2.5_ design concentrations using three methods.

City	No-Guarantee for 5 Days (μg/m^3^)					Guarantee Rate 97.5%					Strict Conditions (μg/m^3^)				
	2020	2019	2018	2017	2016	2020	2019	2018	2017	2016	2020	2019	2018	2017	2016
Harbin	253	191	149	291	162	204	157	153	250	166	204	157	153	250	166
Beijing	160	138	194	247	271	137	145	182	190	273	137	145	182	190	273
Xi’an	191	235	211	304	270	163	227	229	290	217	163	227	229	290	217
Shanghai	108	99	110	99	135	92	104	103	71	135	92	104	103	71	135
Changsha	124	165	147	181	130	131	153	148	173	109	131	153	148	173	109
Guangzhou	56	67	112	82	82	43	76	69	60	61	43	76	69	60	61

**Table 6:** PM2.5 filtration efficiency of different grades of air filters.

Grades	Filtration Efficiency (%)	Grades	Filtration Efficiency (%)
G3	15.0~22.3	F8	62.5~81.3
G4	24.2~26.5	F9	67.3~81.3
M5	25.8~30.2	H10	73.6~82.7
M6	24.1~40.7	H11	81.2~97.0
F7	43.2~61.6		

**Table 7:** The series combination of air filters.

Combination	Filtration Efficiency (%)	Combination	Filtration Efficiency (%)	Combination	Filtration Efficiency (%)
G3 + F7	51.7~70.2	G3 + M5 + F7	64.2~79.2	G4 + M5 + F7	68.1~80.3
G3 + F8	68.1~85.5	G3 + M5 + F8	76.3~89.9	G4 + M5 + F8	78.9~90.4
G3 + F9	72.2~85.5	G3 + M5 + F9	79.4~89.9	G4 + M5 + F9	81.6~90.4
G3 + H10	77.6~86.6	G3 + M5 + H10	83.3~90.6	G4 + M5 + H10	85.2~91.1
G3 + H11	84.0~97.7	G3 + M5 + H11	88.1~98.4	G4 + M5 + H11	89.4~98.5
G4 + M6	42.5~56.4	G3 + M6 + F7	63.4~82.3	G4 + M6 + F7	67.3~83.3
G4 + F7	56.9~71.8	G3 + M6 + F8	75.8~91.4	G4 + M6 + F8	78.4~91.8
G4 + F8	71.6~86.3	G3 + M6 + F9	78.9~91.4	G4 + M6 + F9	81.2~91.8
G4 + F9	75.2~86.3	G3 + M6 + H10	83.0~92.0	G4 + M6 + H10	84.8~92.5
G4 + H10	80.0~87.3	G3 + M6 + H11	87.9~98.6	G4 + M6 + H11	89.2~98.7
G4 + H11	85.7~97.8				

## Data Availability

Not applicable.
